# Catalogue of mitotic chromosome-associated small RNAs in mouse 3T3 cell line

**DOI:** 10.3389/fgene.2025.1559795

**Published:** 2025-10-08

**Authors:** Bishan Ye, Yicong Meng, Le Zhang, Yongzhuo Deng, Xuesong Zheng, Yihang Shen, Xinhui Li, Hua Li

**Affiliations:** ^1^ Jiangsu Province Engineering Research Center of Development and Translation of Key Technologies for Chronic Disease Prevention and Control, Suzhou Vocational Health College, Suzhou, China; ^2^ School of Biomedical Engineering, Shanghai Jiao Tong University, Shanghai, China; ^3^ Key Laboratory of Systems Biomedicine (Ministry of Education), Shanghai Center for Systems Biomedicine, Shanghai Jiao Tong University, Shanghai, China; ^4^ Shanghai Key Laboratory of Embryo Original Diseases, The International Peace Maternity and Child Health Hospital, Shanghai Jiao Tong University School of Medicine, Shanghai, China; ^5^ School of Perfume and Aroma Technology, Shanghai Institute of Technology, Shanghai, China

**Keywords:** small RNAs, mitotic chromosomes, RNA sequencing, mouse 3T3 cells, microRNA

## Introduction

Mitotic chromosomes are essential for faithful genome segregation and play a central role in ensuring genomic stability and the proper generation of daughter cells (Santaguida et al., 2017; Batty and Gerlich, 2019). Nearly two centuries have passed since the initial discovery of chromosomes (Schulz-Schaeffer, 1980) and extensive research has since revealed that mitotic chromosomes are supramolecular complexes of DNA-protein-RNA, containing not only DNA and diverse proteins but also a significant amount of RNAs (Hearst and Botchan, 1970; Uchiyama et al., 2005; Ohta et al., 2010; Meng et al., 2016; Shen et al., 2022; Zhang et al., 2023; Zhang et al., 2024). Mitotic chromosome-associated proteins have been extensively studied using various isolation methods, leading to the identification of thousands of proteins that can be classified into distinct functional categories (Uchiyama et al., 2005; Ohta et al., 2010). Although studies on mitotic chromosome-associated RNAs (mCARs) remain limited, notable progress has been made in recent years. Several studies have demonstrated that certain mCARs play important regulatory roles during mitosis (Rosic et al., 2014; Ma et al., 2022). Our group was the first to perform sequencing-based identification of mCARs using 5′-tag sequencing, leading to the discovery of over one thousand mCARs (Meng et al., 2016). Our follow-up study provides a comprehensive and quantitative catalog of full-length mCARs across multiple species and cell types using high-depth RNA sequencing. This analysis identified a large number of mCARs species, uncovering thousands of enriched and previously unannotated transcripts (Zhang et al., 2024). Another group using a crude isolation method to obtain mCARs also showed that many RNAs are stably maintained and highly abundant on mitotic chromosomes (Shen et al., 2022).

However, the investigation of mitotic chromosome-associated small RNAs (mCASRs) remains lacking. Although previous studies have identified a large number of RNAs that are tightly bound to mitotic chromosomes, they have primarily focused on long RNAs due to limitations in library preparation methods (Meng et al., 2016; Shen et al., 2022; Zhang et al., 2024), thereby resulting in the underrepresentation of small RNAs in these data. Yet small RNAs have diverse subcellular localizations (e.g., cytosol, nuclei) and numerous functions (Dragomir et al., 2018). Notably, small RNAs have been found to associate with mitotic chromosomes and play crucial regulatory roles; for instance, small interfering RNAs (siRNA) have been implicated in chromosomes segregation (Huang et al., 2015). These findings make it tempting to speculate that distinct small RNA species are present on mitotic chromosomes and play important roles there. Thus, identification and characterization of mCASRs represent a scientifically valuable endeavor, with the potential to uncover novel components of mitotic chromosomes and novel functions of small RNAs.

This study addresses this gap by performing high-throughput sequencing of mCASRs from mouse 3T3 cells under three different salt conditions and comparing them with small RNAs isolated from interphase nuclei. Different types of salt buffers can elute components with varying binding affinities to mitotic chromosomes, while maintaining the stability of the core chromosome structure and morphology (Nishino et al., 2012; Meng et al., 2016). Therefore, we investigated mCASRs under different washing conditions to establish a catalogue of mCASRs that are stably and tightly bound to chromosomes.

Overall, for the first time, we provide a dataset cataloguing small RNAs stably bound to mitotic chromosomes in mouse 3T3 cells. The catalogue was generated by stringently aligning and clustering sequencing reads obtained from three independent salt-wash conditions. From an average of >91,000 read clusters, we identified 132 highly and stably associated clusters under all three conditions. Among them, 72 and 6 clusters matched annotated mouse miRNAs and piRNAs, respectively; six were likely TSS-associated RNAs; 48 (36.4%) could not be mapped to known small RNAs, of which 13 were predicted as potential novel miRNAs based on structure and sequence features.

### Value of the data

This dataset constitutes the first comprehensive catalogue of small RNAs tightly associated with mitotic chromosomes in mouse cells, demonstrating that specific small RNAs remain stably bound across distinct experimental conditions. It provides valuable insights into the diversity of chromosomal components and reveals that small RNAs are more widely distributed across subcellular compartments than previously recognized, with notable localization on chromosomes. Our preliminary analysis further transforms the raw data into immediately actionable resource: (1) 132 high-confidence chromosome-bound clusters distilled from three conditions, guiding validation or discovery efforts; (2) annotation of 72 microRNA (miRNAs), six putative Piwi-interacting RNA (piRNA), six putative TSS-associated (TSSa) RNAs and 13 putative novel miRNAs, providing targets for mechanistic assays. Together, this work lays the foundation for future studies on the functions of small RNAs in chromosomal structuring and mitotic process.

## Methods

An overview of our experimental approach is shown in Supplementary Figure S1. In short, demecolcine-trapped mitotic cells were isolated and subjected to hypotonic treatment to lyse the plasma membrane. After crude mitotic chromosomes were isolated by low-speed centrifugation, they were treated with different buffers to wash chromosomes. Then, isolate highly pure mitotic chromosomes (containing RNA) by sucrose gradient centrifugation (5%–60%) and collect the 15%–35% fractions. Then, we isolated the RNAs for small RNA library preparation and sequencing. A novel aspect of our protocol lies in the use of different types of buffer washes to isolate the most stably and tightly bound small RNAs associated with mitotic chromosomes.

### Cell culture

Mouse 3T3 cells (NIH/3T3, ATCC^®^ CRL-1658) were cultured in DMEM (GIBCO, Life Technologies, Carlsbad, CA, USA) supplemented with 10% heat-inactivated fetal calf serum and penicillin-streptomycin (GIBCO, Life Technologies, Carlsbad, CA, USA).

### Mitotic cell synchronization and collection

Cells were cultured until they reached approximately 80% confluency. The culture medium was then replaced with fresh medium supplemented with demecolcine (D1925, Sigma-Aldrich, USA) at a final concentration of 100 ng/mL. Cells were incubated under these conditions for 12 h and subsequently washed with phosphate-buffered saline (PBS). Mitotic cells were dislodged by gentle shaking, collected, and pelleted by centrifugation at 200 *g* for 5 min at 4 C. Mitotic cell purity were quantified by FACS with PI DNA staining. Cells were fixed in 70% ethanol at 4 C, treated with RNase A (0.2 μg/μL) and Triton X-100 (0.1% w/v), stained with PI (20 μg/mL), and analyzed on a BD LSRFortessa. ModFit software was used for cell-cycle modeling, confirming about 95% mitotic purity (Supplementary Figure S2).

### Isolation of mitotic chromosomes and sample peparation under three conditions

Mitotic chromosome isolation was performed as previously described (Meng et al., 2016). Briefly, collected mitotic cells were incubated in hypotonic solution (75 mM KCl) at room temperature for 30 min, pelleted at 600 × g for 5 min, and resuspended in polyamine (PA) buffer (15 mM Tris-HCl, 0.2 mM spermine, 0.5 mM spermidine, 0.5 mM EGTA, 2 mM EDTA, 80 mM KCl, 20 mM NaCl, 0.1 mM PMSF, 1 mg/mL digitonin). After incubation on ice for 5 min, cells were homogenized using a Dounce homogenizer. Subsequent steps were conducted at 4 C. The lysate was centrifuged at 190 *g* for 3 min to yield supernatant S1. The remaining pellet was resuspended, homogenized twice, and centrifuged again to obtain supernatant S2. S1 and S2 were pooled and centrifuged at 420 *g* for 5 min to remove debris, followed by centrifugation at 1,750 × g for 10 min.

To acquire stably and tightly bound small RNAs, we applied differential buffer washes to remove loosely or nonspecifically associated RNAs, thereby enriching for conserved chromosome-associated RNAs. There are three different conditions for washing mitotic chromosome: low salt (LS) buffer (15 mM Tris-HCl, 0.2 mM spermine, 0.5 mM spermidine, 0.5 mM EGTA, 2 mM EDTA, 80 mM KCl, 20 mM NaCl, 0.1 mM PMSF, 1 mg/mL digitonin),which is same as with PA buffer the NaCl concentration is 20mM; high salt (HS) buffer (15 mM Tris-HCl, 0.2 mM spermine, 0.5 mM spermidine, 0.5 mM EGTA, 2 mM EDTA, 80 mM KCl, 220 mM NaCl, 0.1 mM PMSF, 1 mg/mL digitonin)), which is PA buffer supplemented with 0.2 M NaCl; and the buffer A (BA) (15 mM Tris–HCl (pH 7.5), 80 mM KCl, 2 mM EDTA, 2 mM spermine, 5 mM spermidine, 0.1 mM PMSF, and 0.05% digitonin),which is no NaCl. The 0.2 M NaCl concentration was selected based on its ability to preserve protein composition and chromosome morphology while effectively removing non-specific interactions (Meng et al., 2016). Buffer A maintained chromosome size and morphology and effectively removed ribosomal aggregates from chromosome surfaces (Nishino et al., 2012).

For the low salt (LS) sample, the final chromosome pellet was resuspended in low salt (LS) buffer. For the high salt (HS) sample, the chromosome pellet was incubated in high salt (HS) buffer for 25 min on ice, centrifuged at 1,750 × g for 6 min at 4 C, and then resuspended in PA buffer. For the buffer A (BA) sample, the pellet was incubated in buffer A (BA), for 25 min on ice, followed by centrifugation at 1,750 × g for 6 min at 4 C and resuspension in PA buffer.

Chromosome suspensions were layered onto a discontinuous sucrose gradient (5%, 15%, 25%, 35%, and 60% [w/v] sucrose in PA buffer) and centrifuged at 1,100 rpm for 90 min (SW41 Ti rotor, Beckman Coulter, Indianapolis, IN, USA). Mitotic chromosomes were recovered from the 15%–35% sucrose fractions.

### Interphase nuclei isolation

Interphase nuclei (NC) were isolated using the PARIS™ Kit (Ambion, Life Technologies, USA) following the manufacturer’s instructions. Briefly, Freshly cultured cells (∼1 × 10^6^) were harvested and washed once with ice-cold PBS. Cell pellets were resuspended in 500 μL of ice-cold cell fractionation buffer supplemented with 10 μL RiboLock RNase inhibitor (Thermo Fisher Scientific, USA). The cell suspension was gently mixed by pipetting after loosening the pellet via gentle flicking.Samples were incubated on ice for 10 min to facilitate cell lysis. Following incubation, the suspension was centrifuged at 500 *g* for 3 min at 4 C. The supernatant, representing the cytoplasmic fraction, was carefully removed without disturbing the nuclear pellet. To reduce cytoplasmic contamination, the nuclear pellet was washed with the same volume of ice-cold Cell Fractionation Buffer. The pellet was gently resuspended by flicking, followed by re-centrifugation at 500 *g* for 1 min at 4 C. The final supernatant was discarded, and the purified nuclear pellet was retained for RNA isolation.

### RNA isolation, small RNA library preparation and sequencing

RNA from each sample (including BA, LS, HS and NC) was extracted as previously described (Meng et al., 2016). Briefly, the RNA isolation were carried out using Trizol, for RNA precipitation we added a combination of 1/10 volume of 3M sodium acetate (NaAc, pH 5.5), 1 μL of glycogen (20 mg/mL), and an equal volume of isopropanol, which was then incubated at −30 C for more than 2 h. To remove any remaind DNA, the final pellet was resuspended in DEPC-treated water and incubated with DNase I (0.5 U/µl, NEB, USA) and RiboLock RNase inhibitor (0.8 U/µl, Thermo Fisher Scientific, USA)at 37 C for 40 min.The RNA was finally purified using phenol-chloroform-isoamyl alcohol (25:24:1, pH = 6.7) (Sigma,USA)extraction and ethanol precipitation combined with 1/10 volume of 3M sodium acetate (NaAc, pH 5.5), 1 μL of glycogen (20 mg/mL). The precipitated RNA was dissolved in DEPC-treated water for future use. The RNA concentration was determined using a Qubit 3.0 Fluorometer (Thermo Fisher Scientific, USA) with the Qubit RNA HS Assay Kit (Thermo Fisher Scientific, USA).The mitotic chromosome-associated RNAs were validated by RT-qPCR using a positive chromosome-associated RNA marker (U3 RNA).U3 RNA showed higher enrichment on chromosomes compared to mitotic cell total RNAs, with normalized to β-actin (Supplementary Figure S3). Then Small RNA libraries were prepared using TruSeq^®^ Small RNA Library Preparation Kits according to the manufacturer’s protocol (Illumina, USA). In brief, 1 µg of total RNA served as input. After 3′and 5′adapter ligation, RNAs were reverse-transcribed with SuperScript II (Invitrogen, USA), and the resulting cDNA was PCR-amplified for 13 cycles using indexed primers. Amplified libraries were then size-selected to <157 nt (corresponding to inserts <30 nt), and the target bands were excised and recovered.The library quality was verified using an Agilent 2,100 Bioanalyzer. Deep sequencing of the library was performed on an Illumina HiSeq platform using single-end 1x50 sequencing mode.

### Statistical analysis and sequence manipulation tools

The R language (http://www.r-project.org) was used to perform statistical analysis, including hypothesis testing. Sequence manipulation was also performed in R with a variety of R packages used, such as the package “BSgenome.Mmusculus.UCSC.mm10” (version 1.4.0).

## Preliminary data analysis

### Quality control, read alignment and clustering

As described in the Methods, we obtained four samples: three mitotic chromosome samples prepared with different washing buffers—low salt (LS), high salt (HS), and buffer A (BA)—and one interphase nuclei (NC) sample. Raw sequencing data were first processed with FASTX-Toolkit to remove sequencing adaptors and low-quality reads (http://hannonlab.cshl.edu/fastx_toolkit/) (GORDON, G. H, 2010). Reads longer than 30 bp accounted for a very small part of all reads due to the size selection of gel purification in the library preparation and were removed from downstream analysis. Bowtie was then used to map the clean reads generated from the above steps to the mouse genome (version mm10) with default parameters except -v -k and -m all set to 1 (Langmead et al., 2009). Only uniquely mapped reads were used for downstream analysis.

The alignment results for all four samples (BA, LS, HS, NC) as shown in Table 1.

**TABLE 1:** Sequence alignment results. BA–buffer A; LS–low salt; HS–high salt; NC–interphase nuclei.

Sample	Clean reads	Uniquely mapped reads	Multiple mapped reads	Unmapped reads
BA	14,574,286	3,700,987	7,139,185	3,734,114
LS	9,976,026	2,371,135	5,231,165	2,373,726
HS	9,122,280	1,882,901	4,607,028	2,632,351
NC	9,848,377	3,395,127	4,842,266	1,610,984

### Read clustering and positioning

Reads were grouped into the same cluster if the distance between their 5′ ends were less than 15 bp, which is the minimal length of annotated mouse miRNAs. In this study, the highest-frequency read in each read cluster was used to define the position of the read cluster. RPM was used to normalize the read number of each cluster and defined as follows:

$$\text{RPM}=\frac{the\;number\;of\;reads\;in\;a\;read\;cluster}{the\;total\;number\;of\;mapped\;reads}\times 1000000$$

This approach yielded 122,940, 77,275, 75,730, and 96,720 read clusters for BA, LS, HS and NC, respectively. To examine background noise in library preparation, we calculated the length of each read cluster (defined as the distance between 5% and 95% quantile of all the 5′ ends in a read cluster). We found that read clusters longer than 30 bp accounted for approximately 0.2% of all read clusters, demonstrating significant reads aggregation and meeting our expectation of small RNA read distribution.

### Identification of highly and stably associated read clusters

For BA, LS, and HS, it was evident that a larger number of reads in a cluster represented a higher association with the mitotic chromosomes. In each condition, the distribution of RPM showed that <0.3% of clusters accounted for the vast majority of all reads (Supplementary Figure S4). Thus, a cut-off of RPM was set such that the total number of reads in clusters with RPM above the cut-off accounted for >90% of all reads in the condition. The cut-off value and the number of read clusters above the cut-off for each condition are shown in the Supplementary Table S1.

**FIGURE 1:**
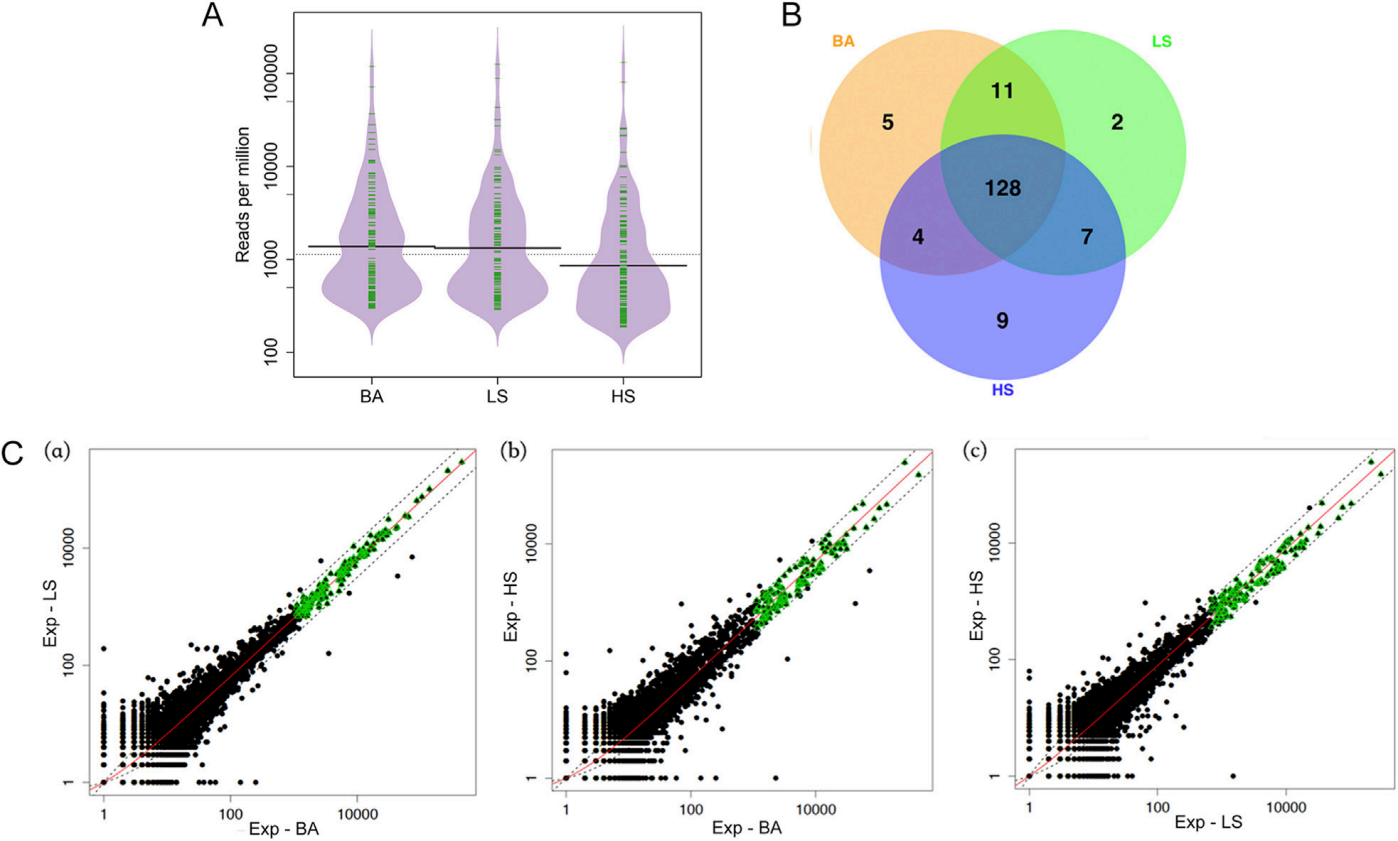
Comparison of mitotic chromosome-associated small RNA in three different conditions. **(A)** The RPM distributions of clusters above the cut-offs. **(B)** The Venn diagram of the three sets of top-ranked 148 read clusters. **(C)** The scatterplot of expression abundance (RPM) between three conditions in mitosis, where the space between dashed lines represents fold changes between 0.5 and 2.

For the read clusters above the cut-offs, we compared the RPM distributions between the three conditions (BA, LS, and HS; Figure 1A). We found that in BA and LS, the average RPM was very close (Solid black line in Figure 1A; p-value >0.05 by Wilcoxon rank sum test), while the average RPM in HS was significantly lower than in the other two conditions (p-value <0.001 by Wilcoxon rank sum test). This indicates that small RNAs of high abundance are more likely to be washed away from chromosomes in a high-salt condition.

The read clusters above the cut-offs are highly consistent across the three conditions. We selected the top-ranked 148 read clusters by RPM in each condition (as shown in Supplementary Table S1) and found 128 read clusters in common (Figure 1B), demonstrating a significant overlap among them (p-value <0.001).

Read clusters with small variations in RPM among different conditions can be considered stably associated with mitotic chromosomes. Here, the definition of “small variation” is listed as follows:

$$R_{\text{BA}‐\text{LS}}={\text{RPM}}_{\text{BA}}/{\text{RPM}}_{\text{LS}},0.5<\;R_{\text{BA}‐\text{LS}}\;<2;$$

$$R_{\text{LS}‐\text{HS}}={\text{RPM}}_{\text{LS}}/{\text{RPM}}_{\text{HS}},0.5<\;R_{\text{LS}‐\text{HS}}\;<2;$$

$$R_{\text{BA}‐\text{HS}}={\text{RPM}}_{\text{BA}}/{\text{RPM}}_{\text{HS}},0.5<\;R_{\text{BA}‐\text{HS}}\;<2.$$

Here, R_BA-LS_, R_LS-HS,_ and R_BA-HS_ represent the RPM fold changes of read clusters between two conditions, with the subscripts denoting the conditions (BA, LS and HS). The RPM scatterplot of read clusters between any two conditions are shown in Figure 1C.

The green dots in Figures 1Ca,Cb,Cc, represent 137, 134 and 139 read clusters, respectively, that are above the cut-offs in Supplementary Table S1 and have small variations as defined earlier. These three sets of read clusters are in high consistency with each other, indicating that a read cluster with small variation between any two conditions is very likely to have a similar RPM in the third condition. The three sets share a common group of 132 read clusters (Supplementary Table S2) that are both highly and stably associated with mitotic chromosomes.

In contrast, most of the 132 clusters show relatively large variation in RPM between each of the three conditions (BA, LS, HS) and condition NC (Supplementary Figure S5); specifically, some of the 132 clusters have very low RPMs in condition NC, indicating their special roles in mitosis. This is consistent with the PCA result showing that the three conditions are in close proximity, while condition NC is farther away from them (Supplementary Figure S6).

### Classification of small RNAs

The miRNA annotation data were retrieved from miRBase (http://www.mirbase.org/; miRBase v21) (Kozomara and Griffiths-Jones, 2014). The Piwi-interacting RNA (piRNA) and transcription start site (TSS) annotation data were retrieved from the UCSC genome browser mouse mm10. Read clusters were identified as miRNAs or piRNAs if they fell into the annotated genomic regions of miRNA or piRNA, respectively. Read clusters were identified as TSS-associated (TSSa) RNAs if they fell into specific regions around TSSs as described in Valen et al., 2011 (−100 nt to +200 nt on the same strand of TSSs; −400 nt to 0 nt on the opposite strand of TSSs) (Valen et al., 2011).

Clusters were classified by comparison to established miRNA, piRNA, and TSSa RNA databases.The majority of 132 read clusters mentioned above were categorized into: miRNA, piRNA and TSSa RNA. First, we compared the 132 read clusters with annotated mouse miRNAs and found that 72 of 132 could perfectly match the miRNA genomic locations. Thus, the 72 read clusters were classified as annotated mouse miRNAs (Supplementary Table S2). As we expected, in all three conditions, the total reads of the 72 miRNAs accounted for more than half of the total reads of the 132 clusters, but the average read numbers showed no significant difference between the 72 miRNAs and the remaining clusters (p-value >0.05 by t-test; Supplementary Figure S7).

piRNA constitutes the largest category of non-coding small RNAs in mammals (Siomi et al., 2011). We compared the remaining 60 read clusters (out of 132) with annotated mouse piRNAs and found 6 with matched genomic positions (Supplementary Table S2). Of the remaining 54 read clusters, there were six located near transcription start sites (TSSs) of six genes, suggesting they likely belong to the TSSa RNA category (Valen et al., 2011). The remaining 48 read clusters (36.4%) could not be mapped to any known small RNA; since they all had very high RPM comparable to the 72 miRNAs, they likely represent novel small RNAs worthy of further validation.

### Prediction of novel miRNAs associated with mitotic chromosomes

Potential novel miRNAs were predicted from the remaining 48 read clusters using miREval2.0 (Gao et al., 2013); expressed sequence tag (EST) annotations from the UCSC mm10 genome browser were also used to support these predictions (Supplementary text, Supplementary Table S3). Among them, 13 read clusters (supported by EST) with hairpin structure, high conservation and shadowing scores were predicted as potential novel miRNAs, which warranted further experiment validation (Supplementary Table S4).

## Data Availability

The datasets presented in this study can be found in the online repository Sequence Read Archive (https://www.ncbi.nlm.nih.gov/sra) under the accession number PRJNA1117372.
