# Supplementary figures and images for: Catalogue of mitotic chromosome-associated small RNAs in mouse 3T3 cell line
Source: Front Genet. 2025 Oct 8;16:1559795. doi: 10.3389/fgene.2025.1559795 (PMC12542727; doi:10.3389/fgene.2025.1559795)

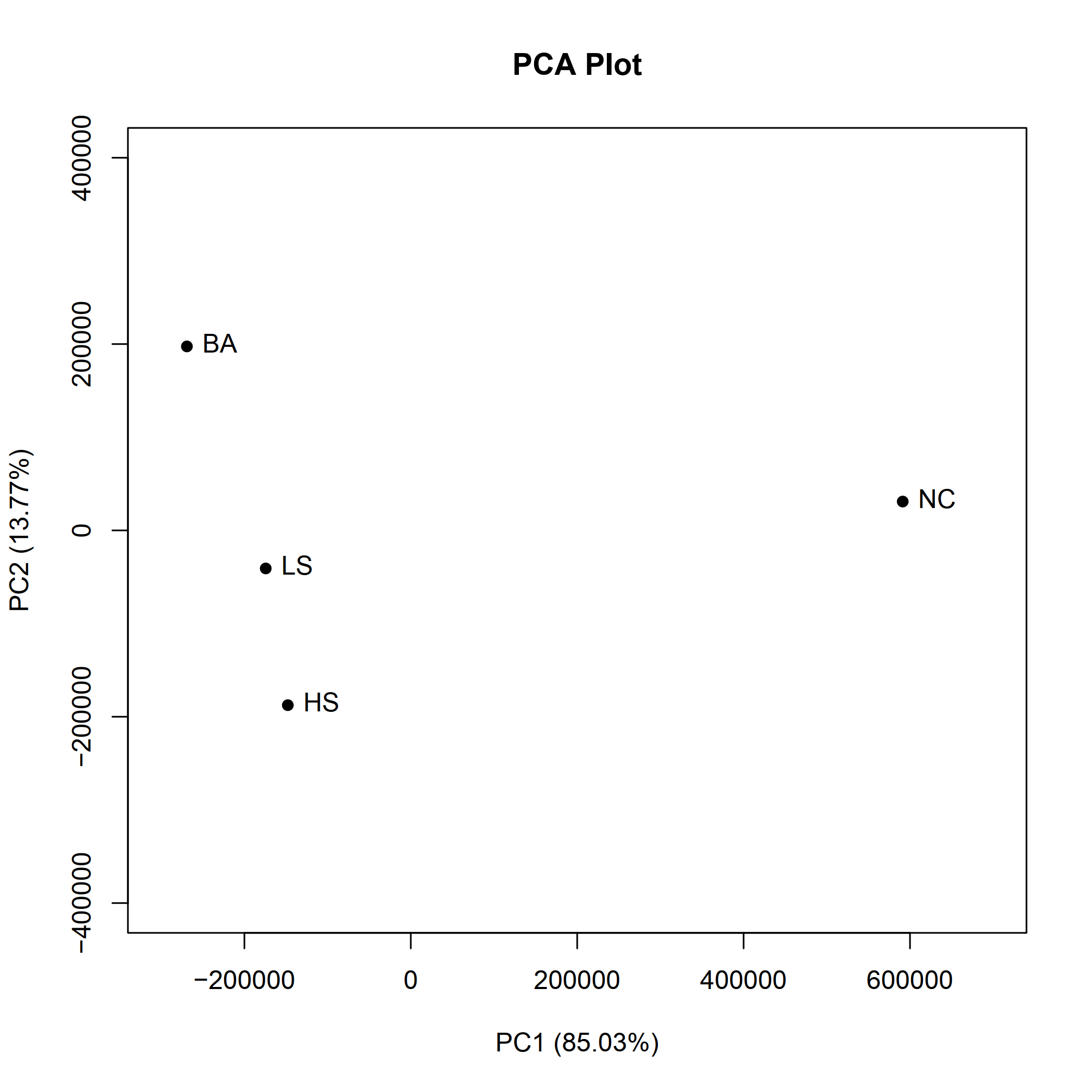

Supplement: Supplementary file 3 [file Image6.tif]

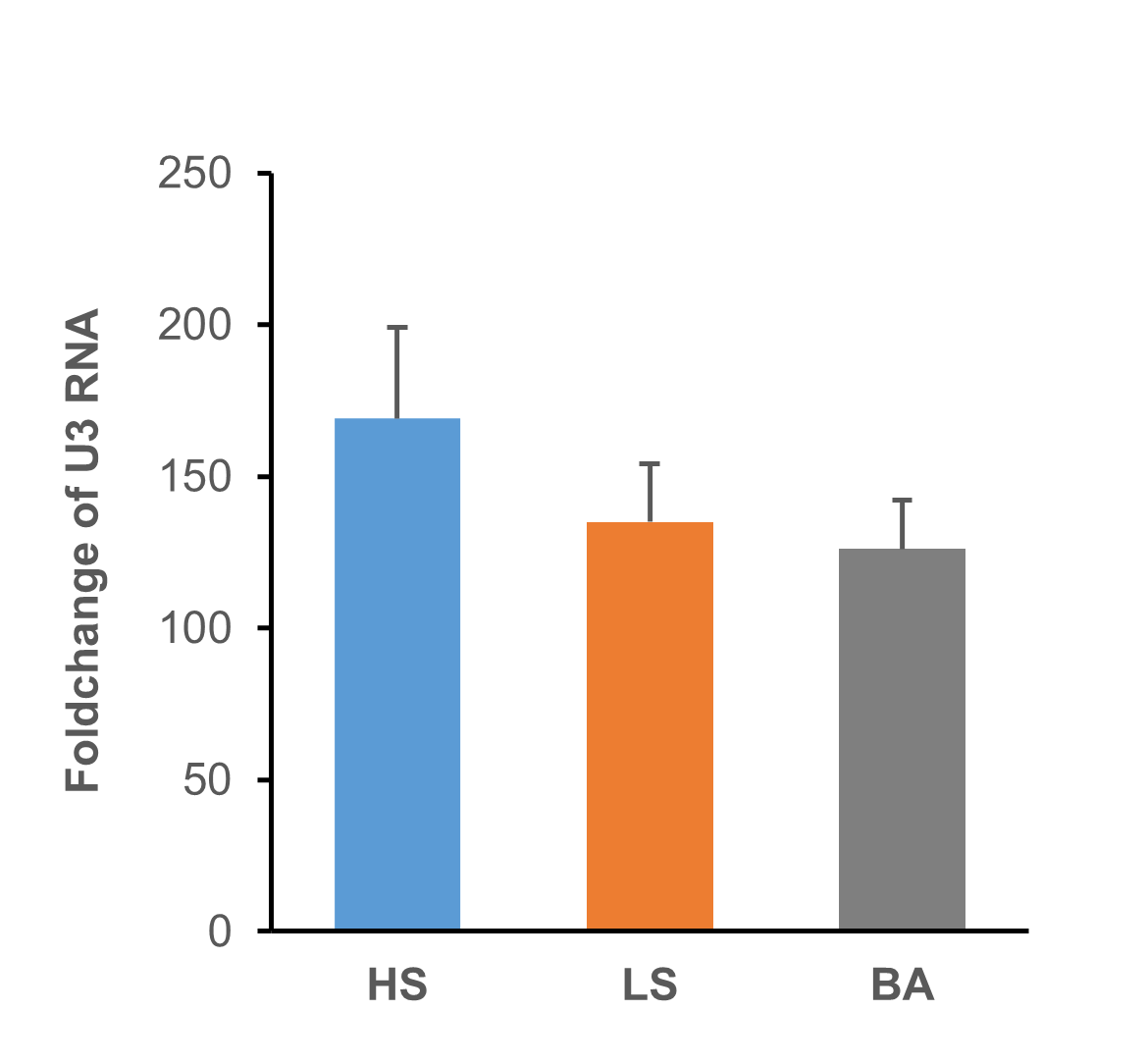

Supplement: Supplementary file 4 [file Image3.tif]

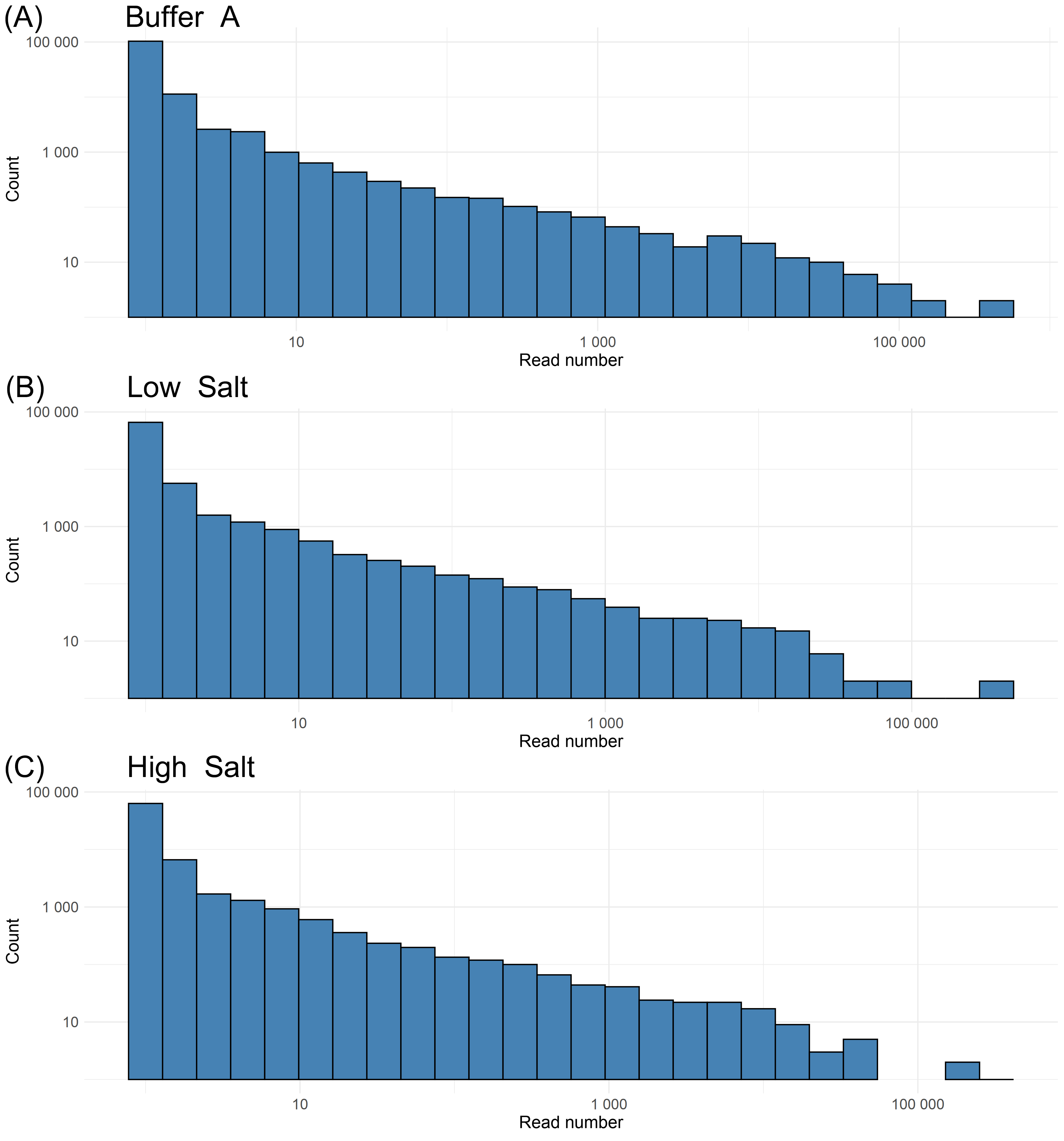

Supplement: Supplementary file 5 [file Image4.tif]

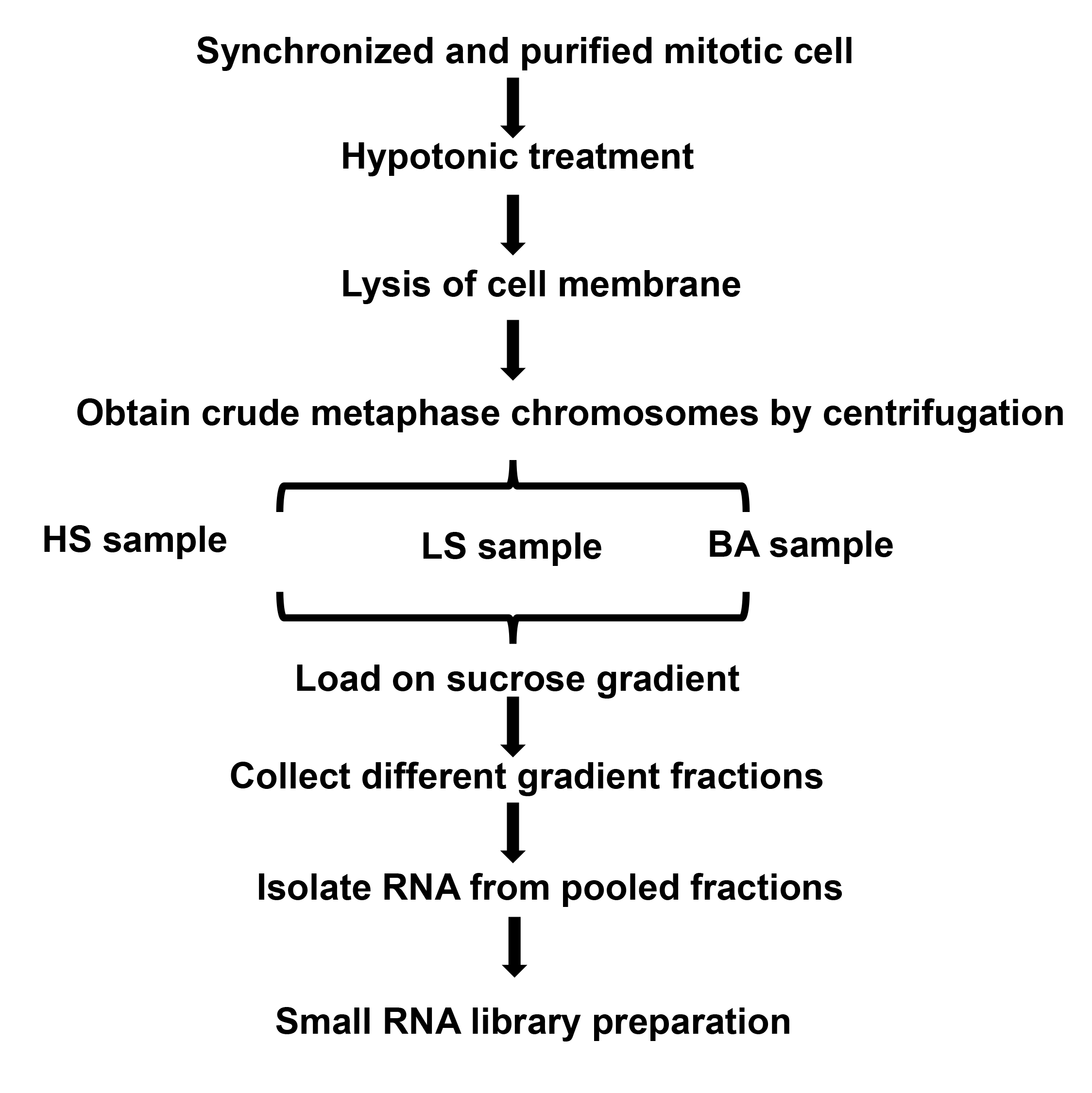

Supplement: Supplementary file 6 [file Image1.jpeg]

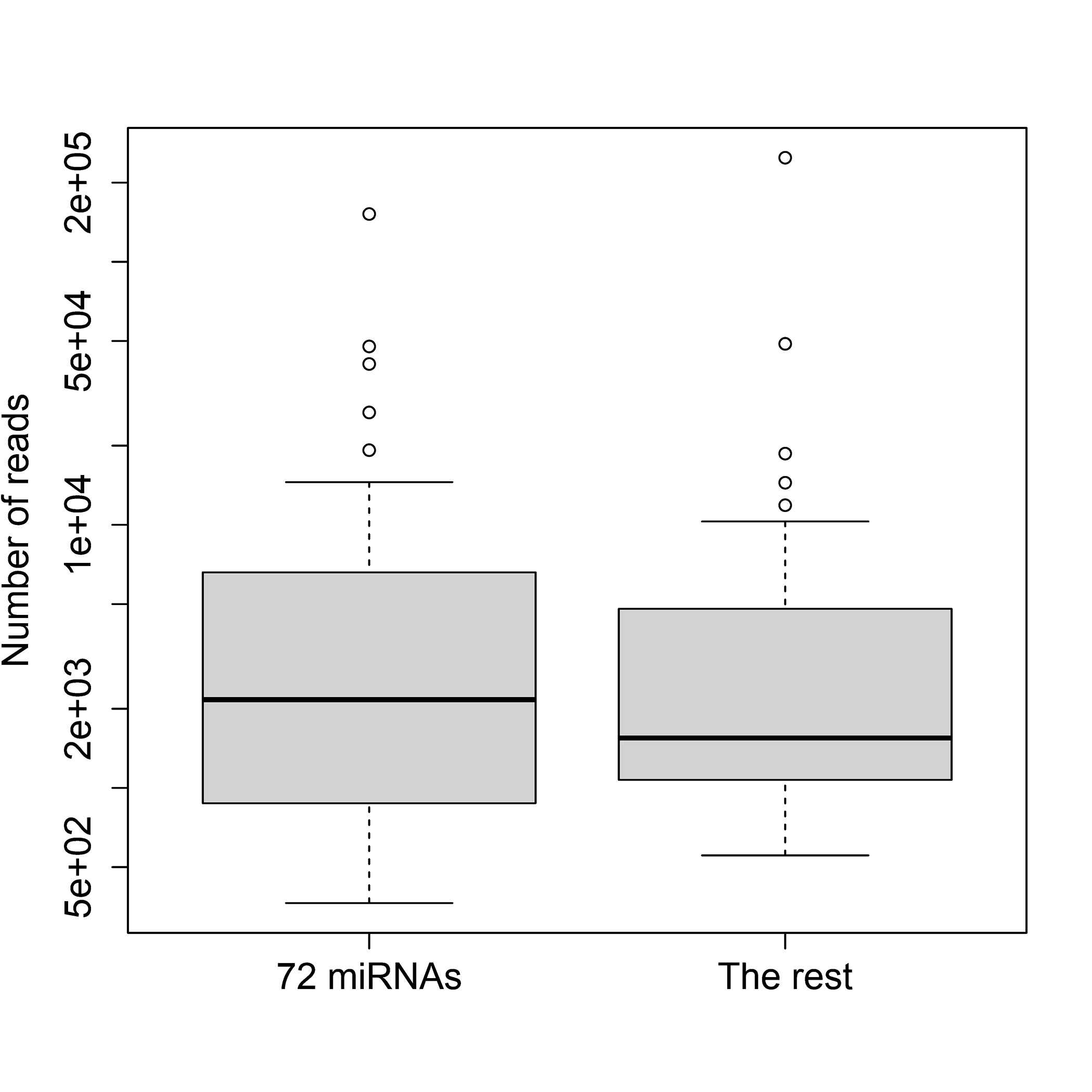

Supplement: Supplementary file 7 [file Image7.jpeg]

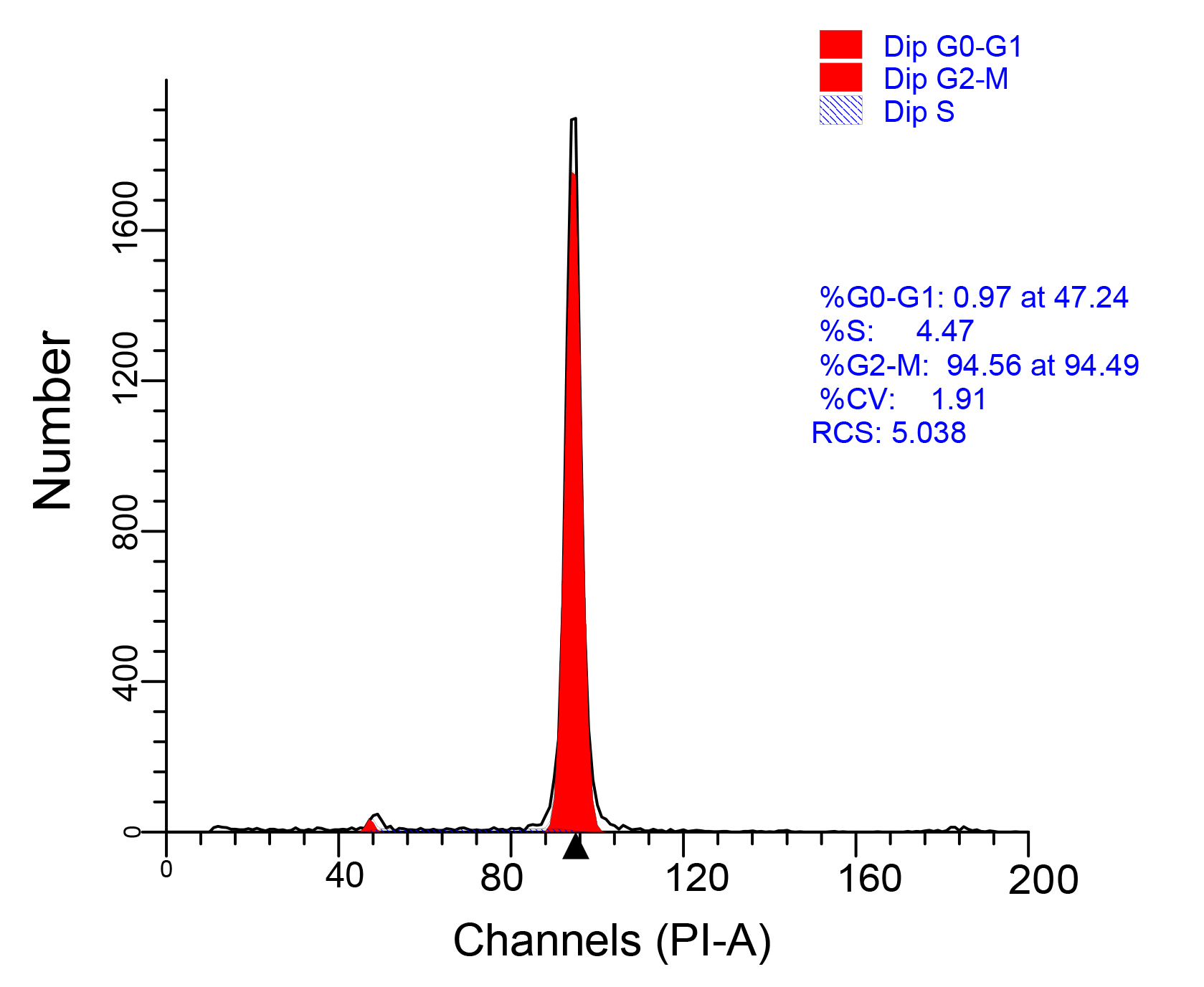

Supplement: Supplementary file 8 [file Image2.jpeg]

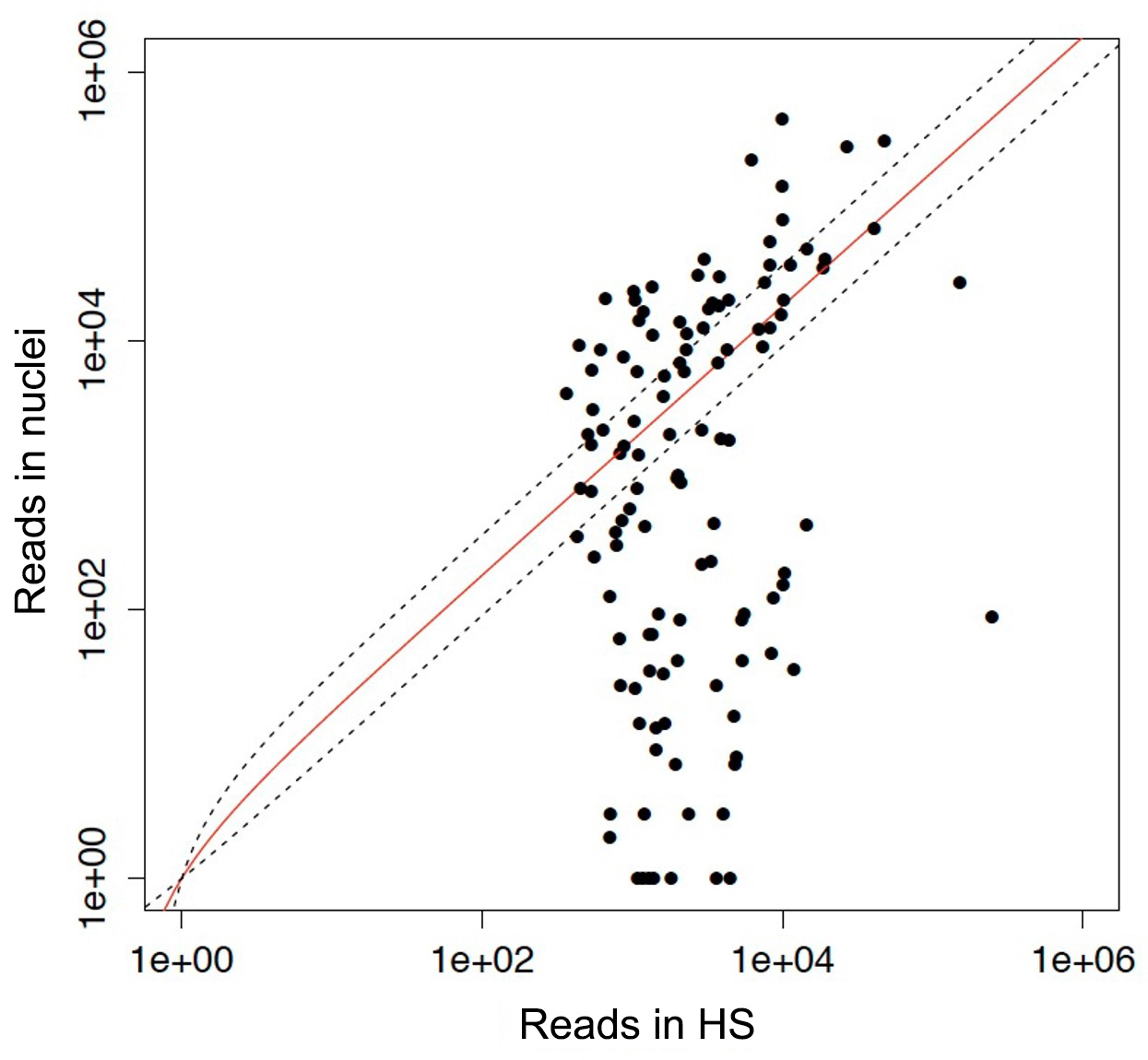

Supplement: Supplementary file 9 [file Image5.jpeg]
