# Supplementary material for: Catalogue of mitotic chromosome-associated small RNAs in mouse 3T3 cell line
Source: Front Genet. 2025 Oct 8;16:1559795. doi: 10.3389/fgene.2025.1559795 (PMC12542727; doi:10.3389/fgene.2025.1559795)
Supplement: Supplementary file 12 [file DataSheet1.docx]

Catalogue of mitotic chromosome-associated small RNAs in mouse 3T3 cell line

# Bishan Ye^1,2,3†^, Yicong Meng^4†^, Le Zhang^2†^, Yongzhuo Deng^2^, Xuesong Zheng^5^, Yihang Shen^3^, Xinhui Li^2*^, Hua Li^1,3*^

1. Supplementary Text

## Prediction of novel miRNAs

We used miREval 2.0 to predict whether the remaining 48 read clusters were potential novel miRNAs (Gao, Middleton et al. 2013). Assuming that the 48 read clusters are all putative miRNAs of 22 nt, we first obtained the sequences including the 22 nt and the flanking regions (±100 nt), and then submitted the sequences (222 nt) to miREval 2.0 for miRNA detection. miREval 2.0 calculated the secondary structure (i.e., hairpin) for miRNA prediction and assessed sequence conservation (e.g., phylogenetic conservation) to aid the prediction. Among the 48 read clusters, more than 90% could form hairpin structure (See Supplementary Table S3); approximately 50% were highly conserved in sequence (the conservation scores derive from the UCSC 60-way vertebrate multiple alignment, including mouse; see Gao et al. 2013 for more details). To further support these predictions, we compared the read clusters with annotated EST and found that 29 clusters could be supported by EST (Supplementary Table S3).

As a result, we identified 13 read clusters with EST support, hairpin structure, high conservation and shadowing scores, which were predicted as potential novel miRNAs worthy of further experiment validation. Additionally, we used MR-microT (Ensembl v84) to predict the target genes of the 13 putative miRNAs and listed the top 10 target genes in Supplementary Table S4 to encourage further investigation by biologists (Kanellos, Vergoulis et al. 2014).

## Reference

Gao, D., Middleton, R., Rasko J. E., and Ritchie, W. (2013). "miREval 2.0: a web tool for simple microRNA prediction in genome sequences." Bioinformatics **29**(24): 3225-3226.

Kanellos, I., Vergoulis T., Sacharidis D., Dalamagas T., Hatzigeorgiou A., Sartzetakis S., and Sellis T. (2014). MR-microT: a MapReduce-based MicroRNA target prediction method. Proceedings of the 26th International Conference on Scientific and Statistical Database Management.

# 2. Supplementary Figure Legends

**Supplementary Figure S1:** Flowchart for the collection of mitotic chromosome-associated RNA. HS, LS and BA stand for high salt, low salt and buffer A, respectively.

**Supplementary Figure S2:** **FACS analysis of the purity of mitotic cells after collection by shake-off.**

**Supplementary Figure S3: U3 RNA enrichment on mitotic chromosomes validated by RT-qPCR. U3 RNA enrichment in three different conditions is compared with U3 RNA in mitotic cell total RNA, which all normalized β-actin.**

**Supplementary Figure S4**: The RPM distributions of read clusters in condition BA, LS and HS. The *x*-axis and *y*-axis have been log-transformed.

**Supplementary Figure S5**: RPM scatterplot of 132 read clusters between HS and NC. The space between black dashed lines represents fold changes between 0.5 and 2.

**Supplementary Figure S6**: Two-dimensional scatterplot of PCA based on all the read clusters from BA, LS, HS and NC.

**Supplementary Figure S7**: Boxplot of read numbers for the 72 miRNAs and the remaining 60 read clusters. The *y*-axis has been log-transformed.

# 3. Supplementary Table Legends

**Supplementary Table S1:** The cut-off values and the corresponding numbers of read clusters.

**Supplementary Table S2:** Genomic coordinates of the common 132 read clusters. 72 read clusters match annotated miRNAs. 6 read clusters match annotated piRNAs. 6 read clusters are putative TSSa RNAs.

**Supplementary Table S3:** Summary of supporting evidence for the 48 read clusters identified as putative miRNAs. The fourth column shows the number of read clusters that form the calculated hairpin structures. The fifth and sixth columns show the numbers of read clusters with high conservation and shadowing scores, respectively.

**Supplementary Table S4:** Sequences and predicted target genes of the 13 putative miRNAs. The first column shows the read cluster number, and the second column lists their sequences. The target genes were predicted using MR-microT (Ensembl v84).

## 4. RT- qPCR Primer

mouse β-actin -R ：CGTTGACATCCGTAAAGACC

mouse β-actin -F：AACAGTCCGCCTAGAAGCAC

mouse U3-F: AAACCACGAGGAGGAGAC

mouse U3-R: ATCAATGGCAGATAGCAGT
